# “Service with open arms”: enhancing community healthcare experiences for individuals with a history of incarceration

**DOI:** 10.1186/s40352-019-0101-1

**Published:** 2019-12-19

**Authors:** Devin Walsh-Felz, Ryan Westergaard, Gabrielle Waclawik, Nancy Pandhi

**Affiliations:** 1 Sutter Santa Rosa Family Medicine Residency, 3569 Round Barn Circle, Suite 200, Santa Rosa, CA 95403 USA; 2 Department of Medicine, University of Wisconsin School of Medicine and Public Health, 1685 Highland Ave, 5th Floor, MadisonMadison, WI 53705 USA; 3 Department of Family and Community Medicine, University of New Mexico, MSC 095040, Albuquerque, NM 87131 USA

**Keywords:** Qualitative, Incarceration, Reentry, Healthcare

## Abstract

**Background:**

The criminal justice-involved population has a higher disease burden than the general population and a high risk of death post-incarceration. However, this group underutilizes healthcare, especially preventive and primary care services. Sixteen in-person, semi-structured interviews were conducted with formerly incarcerated individuals in Milwaukee to explore health impacts of incarceration, barriers and facilitators to healthcare access, and what ideal health service provision would look like following incarceration. Interviews were transcribed, coded, and analyzed using an immersion/crystallization approach.

**Results:**

Overall, people perceived incarceration to have a negative impact on their physical and mental health and expressed dissatisfaction with care in correctional settings. Many faced lapses in care following incarceration, frequently due to insurance challenges.

**Conclusions:**

Participants offered advice for designing an ideal clinic including formal coordination with corrections and provision of additional social services. Staff demeanor that created a welcoming and caring environment was highlighted as an important component and facilitator of care.

## Background

The United States incarcerates more individuals than any other country (Walmsley, 2016), with an estimated 1.5 million prisoners in the state and federal correctional system at the end of 2016 (Carson, 2018). People of color are disproportionately impacted by incarceration (The Pew Charitable Trusts, 2008). At the time of the 2010 U.S. Census, Wisconsin had the highest rate of black male incarceration in the nation (Pawasarat & Quinn, 2013). In Milwaukee County, over half of African American men in their thirties had been incarcerated at some point in their lives (Pawasarat & Quinn, 2013).

There is a growing body of literature around both the health impacts of incarceration and the challenges of accessing care following incarceration. Individuals with a history of incarceration have a higher disease burden than the general population coupled with decreased access to and utilization of healthcare, especially for preventive services and chronic disease management (Binswanger, Krueger, & Steiner, 2009; Katzen, 2011). In addition, incarceration history itself is a risk factor for poor health outcomes (Brinkley-Rubinstein, 2013; Wildeman & Wang, 2017). The first 2 weeks post incarceration are an especially high-risk period, with a risk of death 12.7 times that of the general population (Binswanger et al., 2007). The largest contributor to this heightened mortality risk is drug overdose (Binswanger et al., 2007). Given these risks, much of the literature calls for increasing support for and development of transitional healthcare programs and greater coordination between correctional and community programs in order to support successful reentry (Binswanger et al., 2011; Marlow, White, & Chesla, 2010; Patel, Boutwell, Brockmann, & Rich, 2014; Vail, Niyogi, Henderson, & Wennerstrom, 2017). This call for enhanced patient-centered quality service delivery also highlights the importance of incorporating the advice and perspectives of individuals with a history of incarceration into service design and delivery, a major objective of this study (Laitila, Nummelin, Kortteisto, & Pitkänen, 2018; Omeni, Barnes, MacDonald, Crawford, & Rose, 2014; Phillips et al., 2015; Sharma, Huang, Knox, Willard-Grace, & Potter, 2018).

In a study of newly released prisoners in New Orleans, Louisiana, the authors describe the importance of considering the local landscape when addressing the barriers, desires for services, and health attitudes of formerly incarcerated persons (Vail et al., 2017). This context is especially important given the wide regional variation in criminal justice and healthcare policy that create different structural barriers and influence the environment in which individuals seek or experience healthcare (Vail et al., 2017). While a number of qualitative studies have examined the barriers to accessing healthcare and staying healthy that are encountered by individuals following incarceration, (Mallik-Kane, Paddock, & Jannetta, 2018; Marlow et al., 2010; Vail et al., 2017) there is limited knowledge about the healthcare needs and experiences of individuals with a history of incarceration in Milwaukee, Wisconsin. Milwaukee serves as a valuable case example due to several unique features including heightened racial disparities in incarceration rates and a high rate of insurance enrollment in the reentry population. However, the reentry process in Milwaukee remains insufficiently understood, especially from the perspective of individuals returning to the community.

In addition to adding to the local context, this study expands on topics previously underrepresented in the literature including the impact of incarceration on post-incarceration healthcare, especially as it relates to creating welcoming environments and trusting relationships. We sought to enhance both an understanding of local context and desired healthcare experiences among individuals with a history of incarceration by conducting a qualitative study to better understand perceived health impacts of incarceration, barriers and facilitators to healthcare access, and advice for designing an ideal clinic among individuals with a history of incarceration residing in Milwaukee, Wisconsin.

## Methods

### Context and study eligibility

This study was conducted in partnership with the Salvation Army Medical Clinic, a free clinic providing basic medical services primarily to homeless and uninsured Milwaukee residents. The study was initiated as the Salvation Army Medical Clinic was developing a new service program aimed at improving care for individuals recently-released from jail or prison, seeking to elicit preferences and unique needs that must be met in order to provide optimal care in this context. Individuals with a history of incarceration in jail or prison, age 18 years or older and living in Milwaukee County were eligible to participate. The University of Wisconsin Health Services IRB certified this study as a quality improvement initiative not subject to formal review.

### Recruitment

Recruitment efforts were carried out in neighborhoods disproportionately impacted by incarceration. Posting flyers at grocery stories, public libraries, coffee shops and community organizations yielded 7 participants. Snowball sampling, in which all participants were given interview recruitment handouts to share with acquaintances, yielded 4 participants. Interested participants reached out to the interviewer by phone to schedule an interview, at which point they were screened for eligibility. All individuals who reached out to the interviewer were deemed eligible by eligibility criteria listed above. One interested participant was lost to follow-up. Additionally, on-site recruitment at a community organization that provides post-release services identified 5 more participants. These participants were screened for eligibility, consented and interviewed immediately on-site. Thus, the final sample size was 16 individuals.

### Procedures

The semi-structured interview guide focused on health impacts of incarceration, healthcare needs, healthcare navigation and decision-making following incarceration, experiences with healthcare, barriers and facilitators to care, and advice for care. Questions for the interview guide are shown in Table 1. In an iterative analysis process, after several interviews the guide was refined to provide more focus to questions and/or provide further exploration of ideas that were mentioned in previous interviews. A socio-demographic survey was also developed to gather information on participant demographics, health and healthcare characteristics, and incarceration characteristics. One-on-one semi-structured interviews were conducted between February and April 2018 in a private location by a single interviewer. Interviews lasted approximately 45 min. All interviewees provided verbal consent to be interviewed and audio-recorded. Interviews were transcribed verbatim. Participants were paid $10 from the research team for their participation.

**Table 1:** Selected interview questions

Health Impacts of Incarceration	How do you think incarceration has impacted your health, if it has at all?
Healthcare needs	What are health issues for which you’ve seen a professional?
	I am going to ask you to think back to the first few weeks after you were released from prison.
	What thoughts, if any, did you have about your health?
Healthcare navigation and decision making	Thinking back to the last time you were released from prison or jail: if you have seen a medical professional since then can you tell me about how that happened and what it was like?
	I’ve heard that prior to release from prison/jail you may get help enrolling in insurance and be given information about resources, but you have to make the phone calls and appointments yourself.
	Did this happen for you?
	How do you decide when it’s time to go to a health care provider?
	Have you ever been concerned about how you would be treated at a clinic by staff and providers?
Barriers and facilitators to care	Have you ever decided that you needed care, but didn’t go? Tell me about the reasons you didn’t go.
	If a clinic is hoping to see people within a month of being released from prison/jail, with everything else that is going on in this time period, what might help support people to go to a clinic for a check-up?
Experiences with healthcare	Do you think your incarceration has had any impact on your care at your current clinic?
	Besides having a good relationship with a regular health care provider, are there ways that the clinic makes it easy for you to follow up or creates a space that you want to keep going back to?
Advice for care	If you were the one in charge and could design the perfect clinic for people coming out of incarceration, what would it be like? What would be the most important services for it to provide?
	What advice would you give to staff who are working with individuals who have been incarcerated?

### Data analysis

MAXQDA was used to organize and store coded data. Coding of each transcript was guided by an immersion/crystallization and consensus approach. In this approach, repeated immersions into the data facilitate the emergence of insights and interpretations (Borkan, 1999). Initial coding occurred under domains pertaining to the following areas of interest: health impacts of incarceration, health system navigation, barriers and facilitators, advice for care, and healthcare experiences. Next, in an axial coding process, the data were interrogated to determine relationships between and among codes (Borkan, 1999).

The research team consisted of the lead author who took primary responsibility for coding and analysis, along with a second coder, and a senior researcher both of whom assisted in verifying coding and data interrogation. After codebook revisions, transcripts were revisited to validate patterns. In an iterative process with several crystallization and immersion cycles, codes were split, merged and re-categorized over multiple reviews and as development of the codebook facilitated the identification of new areas of interest.

## Results

### Socio-demographic survey results

Demographics and characteristics of participants (*n* = 16) are described in Table 2. Seventeen individuals were screened for eligibility and determined to be eligible, with 16 individuals completing the interview and one loss to follow-up. As shown, a majority (*n* = 15) were male, identified as African American or Black (*n* = 13), had training or education beyond high school (*n* = 9) and were unemployed (*n* = 11). Incarceration history, including release from prison or jail, time since community reentry, and duration of incarceration, is described in Table 3.

**Table 2:** Participant socio-demographics (*N* = 16)

Characteristic	n (%)
Gender	
Male	15 (94%)
Female	1 (6%)
Race/Ethnicity	
Black/African American	13 (81%)
White	2 (13%)
Latino/Hispanic	1 (6%)
Average Age in Years	47
Range	33–68
Education Level	
Did not complete high school	2 (13%)
High school diploma or GED	5 (31%)
Training or education beyond high school	9 (56%)
Employment Status	
Unemployed	9 (56%)
Unemployed with disability	2 (13%)
Working part-time	2 (13%)
Working full-time	3 (19%)
Housing Status	
Homeless	3 (19%)
Homeless and living with family/ friends	5 (31%)
Renting	7 (44%)
Own home	1 (6%)
Transportation used	
Bus or walk	11 (69%)
Car	6 (38%)

**Table 3:** Characteristics of participants’ most recent incarceration (*N* = 16)

Characteristic	n (%)
Most recent incarceration	
Prison	9 (56%)
Jail	7 (44%)
Time Since Incarceration	
Less than 1 month	1 (6%)
1–6 months	7 (44%)
6–12 months	0 (0%)
1–2 years	3 (19%)
Greater than 2 years	5 (31%)
Length of most recent incarceration	
Less than 6 months	4 (25%)
6–12 months	1 (6%)
12–24 months	4 (25%)
2–5 years	6 (38%)
Greater than 5 years	1 (6%)

Characteristics of health and healthcare utilization, as described in Table 4, reveal that a majority (*n* = 13) of participants were enrolled in insurance at the time of interviews and over half (*n* = 10) were on at least one daily medication at the time of their release.

**Table 4:** Participants’ health and healthcare characteristics

Characteristic	*n* = 16 (%)
Self-rated current health	
Poor	0 (0%)
Fair	5 (31%)
Good	4 (25%)
Very Good	5 (31%)
Excellent	2 (13%)
Health Insurance Status	
Insured	13 (81%)
Uninsured	3 (19%)
Had usual place of care before incarceration	
Yes	6 (38%)
No	10 (63%)
Has usual place of care now	
Yes	10 (63%)
No	3 (19%)
Has appointment to establish care	2 (13%)
Unknown	1 (6%)
Services used since release	
Routine Care	11 (69%)
Mental Health Professional	9 (56%)
Emergency Department	8 (50%)
Urgent Care	5 (31%)
Hospitalized	4 (25%)
None	3 (19%)
On at least 1 daily medication when released	
Yes	10 (63%)
No	6 (38%)
Response to: I work hard at trying to stay healthy	
Strongly Agree	9 (56%)
Agree	6 (38%)
Disagree	1 (6%)
Ever felt that needed care but didn’t go	
Yes	9 (56%)
No	6 (38%)
Unknown	1 (6%)

### Qualitative findings

#### Overview

In order to provide a sense of the range and variation of findings, we use the term “most” to describe experiences in more than half of participants, “several” to indicate an experience or observation in 25 to 50% of participants, and “few” to describe observations that contrast with the majority and are seen in only one or two cases (Pandhi, Bowers, & Chen, 2007). Below we present our results in three sections. First, we describe healthcare experiences during incarceration and how these experiences impacted health and care-seeking post incarceration. Next, we describe barriers and facilitators to accessing care post-incarceration. Finally, we present participants’ advice for post-incarceration primary care services.

Overall, there was considerable variation in how participants navigated the healthcare system, what challenges they encountered and how they made decisions about when to seek care. For most participants, the first episode of care they sought following incarceration was for an acute medical issue. However, the importance of prevention, routine care or chronic disease management were also described as important by most participants. As one participant, released from jail 2 months prior, explained:

*“I was just out there and never went to the doctor, and I had Hep C and didn’t even know it, you know. So that really right there makes me say hey, you got to constantly go, cause you got Hep C and you didn’t even know you had it. You know, so I got to go. Every six months I’m gonna go just to get a checkup and make sure I’m fine.”*

#### Healthcare during incarceration and impact on care-seeking

Most participants perceived a compounding negative effect on their health from the poor healthcare they received during incarceration and the experience of incarceration itself. As illustrated in Table 5, perceived problems with healthcare in correctional facilities included lack of adequate treatment, delayed care, uncaring demeanor, wrong medications given, misdiagnosis, and lack of treatment unless emergent.

**Table 5:** Perceptions of problems with healthcare in correctional facilities

	Illustrative Quote
Lack of adequate treatment	*My leg started to swell up, pus was leaking, they kept me there for an additional 4 more days ‘til the infection spread throughout my whole leg…How do you not send somebody back to the hospital when you see their leg like that?*
Delayed care	*Cause like I say, it could take months in the house of corrections to get seen.*
Uncaring demeanor	*It’s like they don’t care, you know, whether you die in there or not.*
	*It’s like production to them, instead of quality.*
Wrong medications given	*They actually gave this guy the wrong medication ‘cause they didn’t really look at the computer and see what he had. The guy went into a seizure and a coma.*
Misdiagnosis	*I mean, you could have a heart problem they’ll tell you you have the flu and later on down the line you find out something else is wrong.*
Treatment lacking until emergent	*Before they really even take actions, full actions, you got to pass out or, you know it shouldn’t have to get to that point.*

Most participants felt that incarceration had a negative impact on their health, with half of individuals describing adverse effects on mental health. One individual who had been incarcerated multiple times, having most recently spent over 2 years in prison, explained:

*“The greatest health epidemic in prison is mental. You know, because you destroy a person’s spirit, you destroy their sense of worth, you know, like I’m not a man like you, I’m not a woman like you now because I’m locked up.”*

A few individuals felt that incarceration had no impact on their health and a few described incarceration as preserving health or even improving some aspects of health:

*“So I was healthier and my body had a chance to mend without the drugs, without the cigarettes, without the uh, the demands of rent and food and a job and a family or a woman, you know.”*

Most interviewees said that their negative healthcare experience during incarceration did not hinder their future care seeking and several said it actually encouraged them to seek care following incarceration. An individual who spent 5 years in prison explained:

*“After you do a little time or whatever, it’s not the best healthcare system in the world. Of course a lot of complaints and things of that nature are overlooked. So you really, I mean you kind of say to yourself, oh I get to see a real doctor, or I get to go to a real facility and so those were my main two things. To see somebody more professional in my opinion, and to just acquire healthcare.”*

Nearly half of people mentioned the need for more resources about care options as a part of pre-release planning or the potential utility of promoting the availability of services to people prior to their release from prison or jail.

#### Barriers and facilitators to healthcare system navigation following incarceration

There was variation in how participants navigated the healthcare system after incarceration and what challenges were encountered. Commonly mentioned challenges and barriers were related to insurance, cost and transportation. Important facilitators included the role of community organizations and word of mouth.

While most participants did not have a regular place of care prior to incarceration, at the time of participation in interviews, most participants had active health insurance and a regular place of care. However, a few had never received primary care services outside of incarceration, and those who were receiving care still faced barriers and lapses in care at times. These barriers and lapses were primarily due to insurance or financial concerns and lack of transportation. While several people had no trouble enrolling in insurance, lapses in insurance were common, with nearly half of participants experiencing a gap in insurance coverage and several describing lack of insurance as a reason for not seeking care. Insurance challenges mentioned by participants included difficulty maintaining enrollment, employment related insurance gaps, lack of insurance and underinsurance. Without insurance, participants generally expressed lapses in care due to financial concerns. One individual who established care 7 years after his release from jail shared:

*“I let my asthma get worse and worse and worse because when I think of doctor…I think copay. I think I just don’t have it. I don’t have the copay.”*

Several people mentioned the important role of community organizations in navigating the healthcare system through providing support for insurance enrollment and/or finding a clinic and doctor. Additionally, most mentioned word of mouth as an important aspect of healthcare navigation. Participants described using word of mouth to assist them in figuring out a wide range of navigation factors including what doctor to go to, hospitals to seek or avoid, treatment approaches, eligibility for services and existence of helpful services. One person who spent a year and a half in the House of Corrections described the importance of word of mouth during incarceration that ultimately helped him seek treatment for Hepatitis C once he was released:

*“So basically, a lot of information you get is like from people that’s in there among people that you talk about, you know, you talk around, and it’s like people just, other inmates just help other inmates, you know, you got older inmates, you got younger inmates, and everybody’s just trying to help each other out.”*

Others described post-incarceration word of mouth as an important factor in seeking treatment. A 41-year-old man released from jail 2 months prior who was actively seeking mental health care explained:

*“So I check around with other people that, a lot of people that I deal with or that I associate with have had the same problems I’m having. You know what I’m saying, from the medication side effects and things of that nature. So I take their advice and see if the places they’re going to is really going to help me like it’s helping them.”*

Word of mouth was also described as helpful in finding a doctor or clinic, and was powerful both years after information was shared as well as more immediately:

*“I remember one of my friends, like, from 2011, she always spoke about her [doctor]…So I remembered that name, looked her up, and I went to the urgent care and she told me to come to her office, and I went. It was just really word of mouth I guess.”*

*“One day I was with my buddy and he’s like ‘I’m going to urgent care and gonna walk in,’ and I just happened to go in with him, and was like, you got insurance card why don’t you try this.”*

#### Advice for transitional care

When participants were asked to describe the ideal clinic for people coming out of incarceration, they commonly talked about such a clinic providing additional social services, addressing mental health, and fostering a friendly and welcoming environment. Table 6 displays participants’ specific advice for such a transitional clinic with illustrative quotes.

**Table 6:** Participants’ advice for an ideal clinic

	Illustrative Quote
Communicate regularly	*Make somebody believe that they’re worth something and that you care, so I mean a lot of guys don’t have cell phones right away, so it’s hard to get in contact with them, or they might not even have an address. But if they have an address or a cell phone or something, do a follow up… just say yeah Mr. [name] we’re just calling, checking up on you, you know we will be seeing you soon, how are you doing today? Doing out there? Is there any resources that we can provide for you?*
Offer timely care	*If it was my clinic you’d come right in, you’d get seen that day, you would get checked up that day.*
Take concerns seriously	*Listen to what’s going on, what he has to say to you, and really take the situation of the care of the health serious…A lot of times they feel like ‘oh inmates just say anything, just to get off the dorm just to go here, just to do this,’ but it’s not like that all the time. Some people are really sick in there. And I mean, just take it serious, really though. Take the inmate serious, that’s all.*
Coordinate with corrections	*I was thinking like maybe they should have gave you like resources or options to go like to free clinics, where people with no insurance could like, maybe they could send a person there instead of just starting them right out in the water.*
Prioritize mental health	*When you work with these guys mainly stay focused on their mental…They need more mental help than they do probably physical. And they really do.*
Offer social service support	*You have the basic necessities…where you gonna live, housing, of course employment, support groups.*
Provide incentives	*Maybe offering something like, you know, if you come we have meals, hot meals served, you know with your visit. Um, some sort of incentive I guess.*
Provide a caring and non-judgmental environment	*Just listen and try to see from their perspective first.*
	*I think that the more comfortable the facility, the more inviting a facility, for the staff to really be trained to…be motherly almost, to be comforting, to show that that comfort and acceptance is probably the most important thing.*
	*Don’t treat them like they’re institutionalized…people who are institutionalized and people who have been in the system for so long, they’re used to a certain type of personality. They’re used to overseers. You know what I mean? Kind of treat people softer, maybe use humor.*
	*No discrimination against you…it would be service with open arms. We there to take care of you, no matter what your situation is. We understand that you just getting out, but we are here to help you.*
	*Don’t judge the book by the cover. Everyone has their own story and every story is different. How you go about dealing with the situations is this, listen to the problem from the mouth of the person who has the problem.*

Most participants mentioned the importance of providing additional social services including support for employment, transportation, housing, and food. Having multiple services available on-site was described as ideal, otherwise it was recommended to provide referrals or informational brochures. Several individuals mentioned the importance of prioritizing mental health as part of an initial assessment and having mental health services available, primarily counseling or therapy.

Friendly, respectful service from clinic staff and creation of a non-judgmental, empathetic environment were also highlighted as important aspects of care. Examples of good care experiences that were described included taking concerns seriously, listening, providing thorough care, providing appropriate referrals, personalizing care and perceptions of staff as friendly and caring. Generally, individuals did not feel that their history of incarceration impacted the care they received at a clinic, and most expressed that they never had concerns about how their doctor would treat them. Several interviewees emphasized an overall perception of providers as caring and passionate about helping people. A 51-year-old man who had recently been incarcerated in prison for over 3 years shared:

*“Yeah, they open armed because I guess, a lot of them do love their profession. And most importantly, they love helping others, and they want to see people healthy. So they usually come with the best possible care, and information.”*

However, a few individuals did express concerns about feeling out of place or stigmatized. One individual, a 61-year-old who had spent 11 months in jail, describes feeling out of place in a clinical setting:

*“It’s just when you go to doctors and dentists and you know, business places or important places, you feel like you don’t fit if you’re not clean and nice and decent you know, so…You feel out of place, everybody else is nice and clean and fresh and laughing and optimism and happy and joyful and you down and out and smell yourself and god damn I gotta get outta here. And you feel like everybody’s talking about you and looking at you.”*

A 31-year-old who had spent over a year in prison and had never accessed healthcare outside of incarceration described concerns about stigma and what doctors might think after long lapses in care:

*“That’s [discrimination] always in the back of your head because people think, um, everybody who goes to prison is a bad person, but that’s really not the case, because we are human beings, and human beings make mistakes.”*

*“It’s like going into something that you haven’t been for a long time. That’s like, you haven’t been to church in 10, 15 years but then all of a sudden you in church again so you kind of worry like, what are people going to think? Or, ‘hey Mr. [name], where you been at? Why haven’t you came to see me? Or seen a doctor in 10 years?’…so that’s what I’m worrying about, the doctor scolding me.”*

## Discussion

This study adds to the small but growing body of literature that seeks to explore the healthcare desires and experiences of individuals with a history of incarceration. We found that participants experienced incarceration as having a primarily negative impact on their health, which was compounded by the perception of inadequate healthcare during incarceration. In contrast, after being incarcerated, many of our participants felt that they were receiving good quality care, which they described as providers who communicated regularly, listened, and took concerns seriously. We also found that while individuals expressed the importance of having a regular place of care and seeking preventive care or chronic disease management post-incarceration, lapses in care were common and individuals faced multiple barriers to accessing care. Our participants also saw the opportunity to improve the quality of care through the provision of additional services not traditionally offered in the clinical setting such as resources for jobs, food, transportation and housing.

In addition to the perceived negative impact of incarceration on health, healthcare experiences during incarceration were generally considered unsatisfying, with a lack of trust in correctional healthcare. Other studies have documented similar negative experiences with correctional healthcare (Vail et al., 2017). An overall distrust of the medical field has been previously identified as a barrier to seeking care in this marginalized population (Howerton et al., 2007; Marlow et al., 2010). In this study however, the trend was that poor care experiences during incarceration may have encouraged care seeking after community reentry for individuals who did not feel that their health needs were adequately addressed during incarceration. Taking into consideration both the negative health impacts of incarceration as described by participants and the perceived lack of quality care in correctional institutions, one implication of our findings is to suggest that providers may want to ask individuals how they feel incarceration has impacted their health and if they have health concerns that were not addressed while incarcerated.

While individuals expressed a mistrust with the correctional system, they did express a desire for increased coordination between correctional institutions, clinical services and community organizations. The importance of pre-release and post-release service coordination is also supported by other literature (Wang et al., 2010). Our participants echoed the need for programs and clinics specifically tailored to individuals coming out of incarceration (Marlow et al., 2010; Vail et al., 2017; Wang et al., 2012). These findings encourage clinics that are trying to optimize care for individuals with a history of incarceration to strengthen their referral relationship with the Department of Corrections and to develop “inreach” programming in which a representative from the clinic would provide information about services at correctional institutions. Furthermore, results revealed that word of mouth and referrals from community organizations were particularly powerful in supporting health system navigation. Other successful models of transitional care have shown that Community Health Workers with a history of incarceration improve the reach of their services and are an invaluable asset when it comes to peer support, mitigating stigma (Fox et al., 2014), and building trusting relationships Wang et al., 2010). While substance use treatment did not emerge as a major theme from our study, this is another area for which coordination between correctional institutions and community organizations is critical, especially given the heightened risk of death from overdose following incarceration (Binswanger et al., 2007). Given the multitude of opinions on this topic in our study, we feel it is important to include the perspectives of individuals who would be served by transitional care programs in order to guide service delivery in a responsive manner that engenders the appropriate level of trust and confidentiality.

Caring provider attitudes and behaviors were emphasized when describing good care experiences and in providing suggestions for an ideal clinic. While many mentioned the importance of a non-judgmental, empathetic environment, most healthcare experiences in clinics outside of the correctional setting were described positively and individuals often described healthcare providers in high esteem. A previous study looking at perceptions of community healthcare among parolees found a caring professional demeanor to be an important facilitator of healthcare access, facilitating access even when barriers were present (Marlow et al., 2010). The study provided frameworks for providers to incorporate caring and empathetic behaviors in their practice by starting encounters with listening, emphasizing equal partnership and utilizing motivational interviewing (Marlow et al., 2010). Other ways providers can show care, as described by participants in our study, are to communicate regularly with patients, express the importance of taking concerns seriously, and spend time listening. The contrast in perceptions of care between the correctional and community environment and the emphasis on caring staff demeanor as an important component and facilitator of care highlight the importance of “stigma informed care” in this population. This idea reflects the sentiment that incarceration can be a dehumanizing experience and that individuals deserve care that acknowledges this trauma through an intentional focus on humanity, dignity and compassion.

When considering stigma in this population, there are multiple ways in which it affects health and successful reentry. Stigma directly impacts mental health and self-esteem (Wicks, 2017) along with the ability to secure safe housing and employment (Wicks, 2017; Visher et al., 2011). In turn, securing housing is a critical step in societal reintegration and stigma mitigation (Wicks, 2017). Additionally, self-transformation supported by a sense of meaning and purpose, and social and emotional supports are also found to be cornerstones of successful reentry (Hlavka et al., 2015). In the context of managing multiple priorities including food, housing, employment and re-establishing social ties, accessing healthcare may not be a top priority (Ramaswamy, Upadhyayula, Chan, Rhodes, & Leonardo, 2015). Our participants similarly described competing priorities as well as a desire to access multiple services in a single setting, and suggested the provision of incentives including meals or snacks as a way to encourage healthcare visits. The finding highlights the need to consider social determinants of health in the clinical setting and to offer support with basic necessities such as food, employment, transportation and housing in the form of referrals or on-site social work. Another possible implication of this finding is for clinics hoping to optimize services to engage in outreach in which providers deliver on-site services in locations where other post-incarceration services are being provided. This has the potential to co-locate services as participants in our study desired, and to facilitate relational capacity building with community organizations as well as individuals seeking services. Home visits or telehealth are other innovative service delivery options with potential to improve access, especially amongst individuals for whom transportation is a major barrier.

Our study is subject to several limitations. Convenience sampling and recruitment through community organizations may have resulted in a sample with greater connection to services. Our study participants were predominantly black males and therefore may not be fully representative of the variability of post-incarceration experiences by other demographic groups. However, the black male population is disproportionately affected by incarceration, (Bonczar, 2003) and therefore understanding experiences in this group is of singular importance. Our low recruitment of women may reflect the lower rates of incarceration of women compared to men. Further exploration of the experiences of women with a history of incarceration would be a valuable future research direction. Despite these limitations, this study provides several considerations for actionable findings for clinics that are serving individuals with a history of incarceration.

## Conclusion

In summary, the period following release from prison or jail is a high-risk period for individuals who face complex challenges and vulnerabilities. Participants in this study offered advice for designing an ideal clinic including formal coordination with corrections and co-location of multiple services, including services not typically provided by healthcare organizations such as employment, food, housing and transportation resources. The importance of staff demeanor in creating a welcoming and caring environment was highlighted as an important component and facilitator of care. Continuing to incorporate the voices of individuals with a history of incarceration is critical when seeking to deliver compassionate, responsive, high quality healthcare services to this highly vulnerable group.

## Data Availability

Data sharing is not applicable to this article as no datasets were generated or analyzed during the current study.
